# Supplementary material for: Flexible versus Rigid Bronchoscopy for Tracheobronchial Foreign Body Removal in Children: A Comparative Systematic Review and Meta-Analysis
Source: J Clin Med. 2024 Sep 23;13(18):5652. doi: 10.3390/jcm13185652 (PMC11433179; doi:10.3390/jcm13185652)
Supplement: Supplementary file 1 [file jcm-13-05652-s001.zip › Table S1.pdf]

Supplementary Table S1. The search query employed in the literature review, adjusted per each database

Database	No	Search Query	Results
**PubMed [Date of search: March 1, 2024]**			
	#1	Flexible[tiab]	149104
	#2	Rigid[tiab]	70154
	#3	Bronchoscop*[tiab]	32976
	#4	"foreign body"[tiab]	30124
	#5	Children[tiab] OR "Child"[Mesh] OR infant[tiab] OR "Infant"[Mesh] OR "Child, Preschool"[Mesh] OR pediatric[tiab]	3235383
	#6	#1 AND #2 AND #3 AND #4 AND #5	129
**Scopus [Date of search: March 1, 2024]**			
	#1	TITLE-ABS-KEY (Flexible)	744268
	#2	TITLE-ABS-KEY (Rigid)	326798
	#3	TITLE-ABS-KEY (Bronchoscop*)	76501
	#4	TITLE-ABS-KEY ("foreign body")	86970
	#5	TITLE-ABS-KEY (Children) OR TITLE-ABS-KEY (infant) OR TITLE-ABS-KEY (pediatric)	4436042
	#6	#1 AND #2 AND #3 AND #4 AND #5	230
**Web of Science [Date of search: March 1, 2024]**			
	#1	AB=Flexible	470682
	#2	AB=Rigid	202478
	#3	AB=Bronchoscop*	21087
	#4	AB="foreign body"	18058
	#5	AB=Children OR AB=infant OR AB=pediatric	1599636
	#6	#1 AND #2 AND #3 AND #4 AND #5	100
**Cochrane Library [Date of search: March 1, 2024]**			
	#1	Flexible	9474
	#2	Rigid	3489
	#3	Bronchoscop*	3933
	#4	"foreign body"	1669
	#5	Children OR infant OR pediatric	243626
	#6	MeSH descriptor: [Child] explode all trees	81477
	#7	#5 OR #6	243626
	#8	#1 AND #2 AND #3 AND #4 AND #7	0
**Google Scholar [Date of search: March 1, 2024]**			
	With all of the words	Flexible rigid child	-
	With the exact phrase	Foreign body	-

	With at least one of the word	Bronchoscopic bronchoscopy bronchoscopically bronchoscope	-
	Total	As per the guidelines, only the first 200 records were screened	200
