# Supplementary figures and images for: Flexible versus Rigid Bronchoscopy for Tracheobronchial Foreign Body Removal in Children: A Comparative Systematic Review and Meta-Analysis
Source: J Clin Med. 2024 Sep 23;13(18):5652. doi: 10.3390/jcm13185652 (PMC11433179; doi:10.3390/jcm13185652)

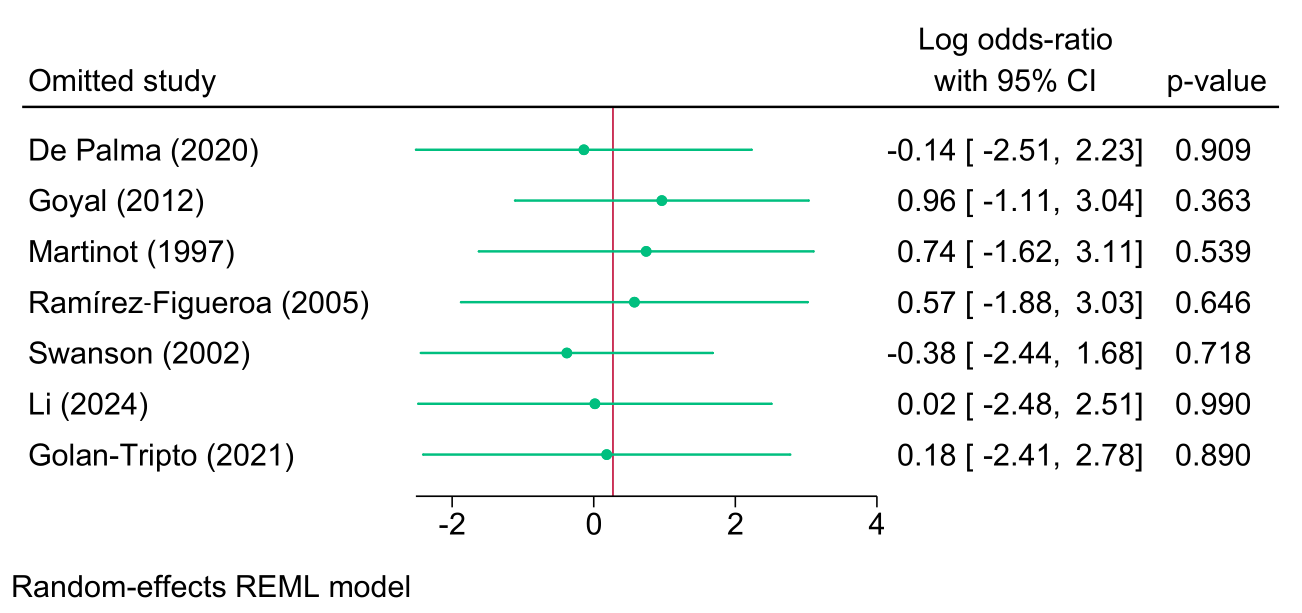

Supplement: Supplementary file 1 [file jcm-13-05652-s001.zip › Figure S1.tiff]

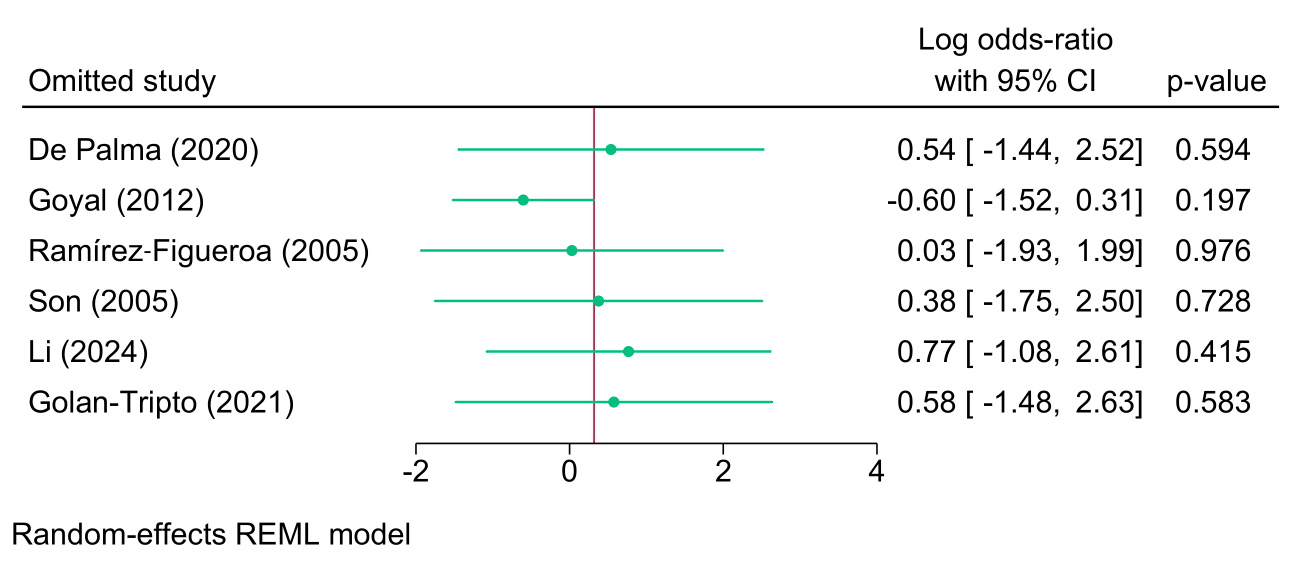

Supplement: Supplementary file 1 [file jcm-13-05652-s001.zip › Figure S2.tiff]

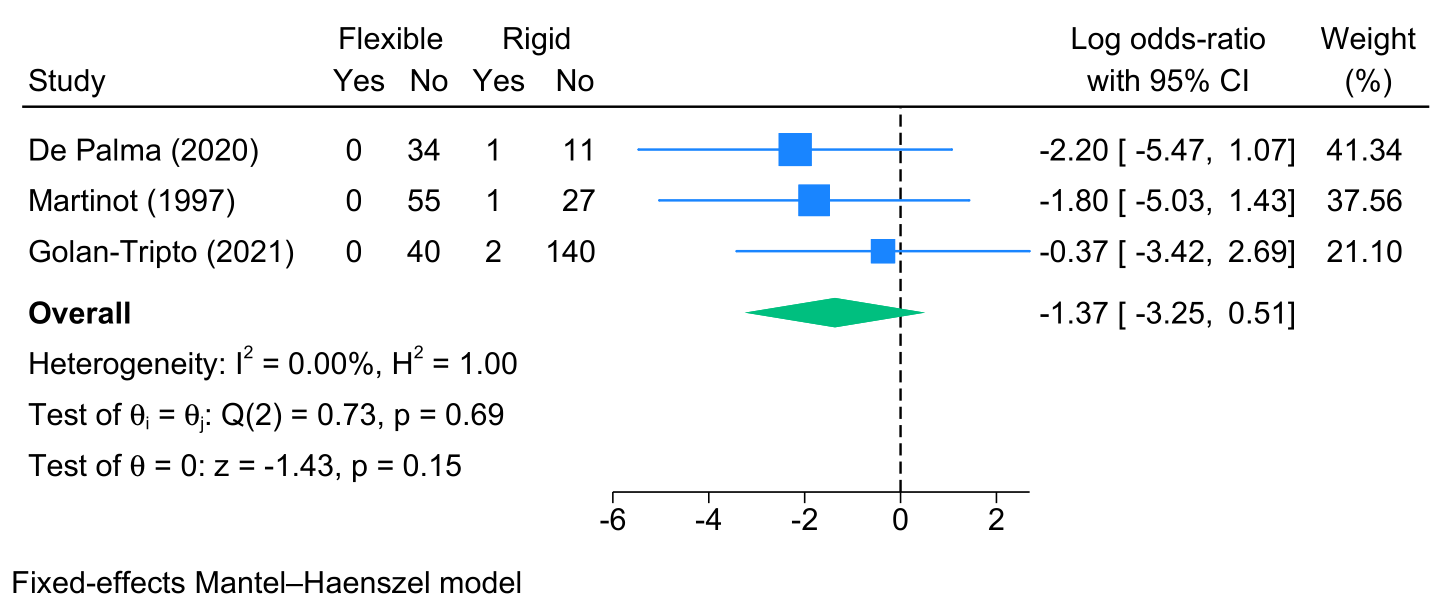

Supplement: Supplementary file 1 [file jcm-13-05652-s001.zip › Figure S3.tiff]

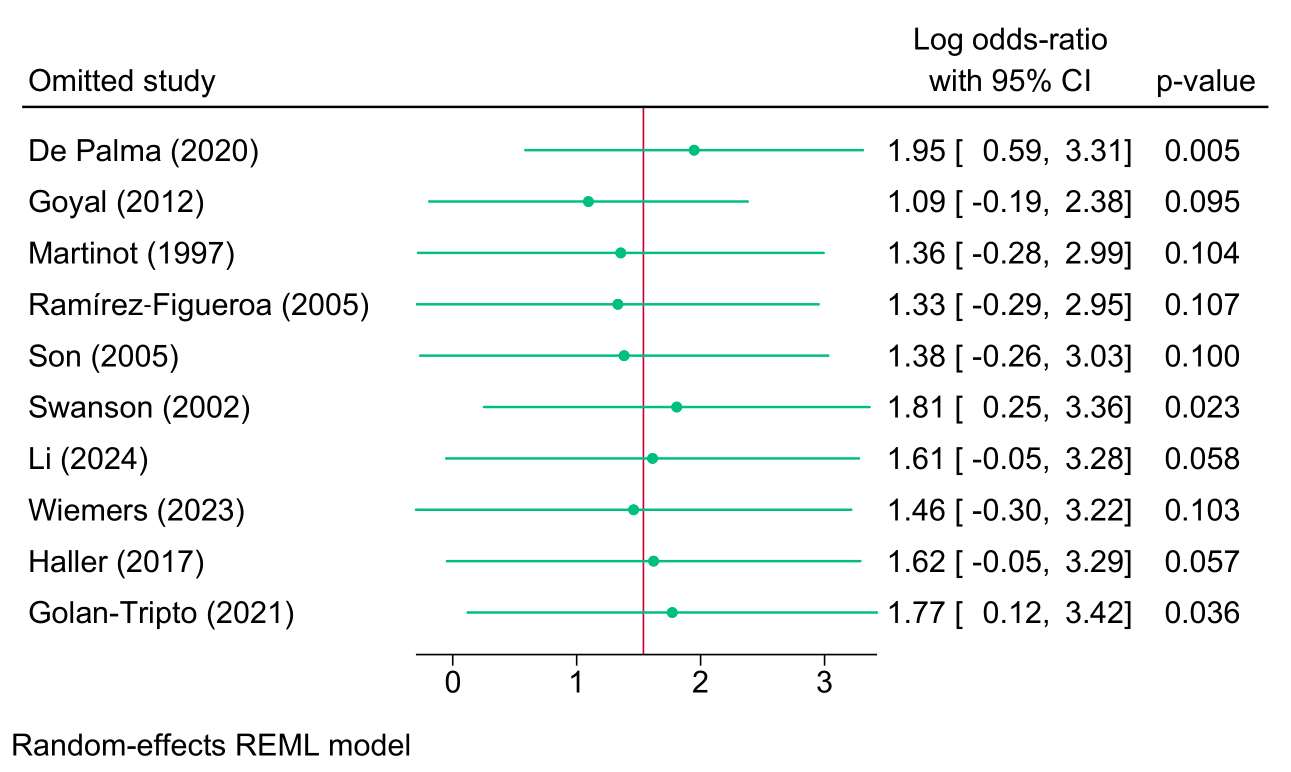

Supplement: Supplementary file 1 [file jcm-13-05652-s001.zip › Figure S4.tiff]

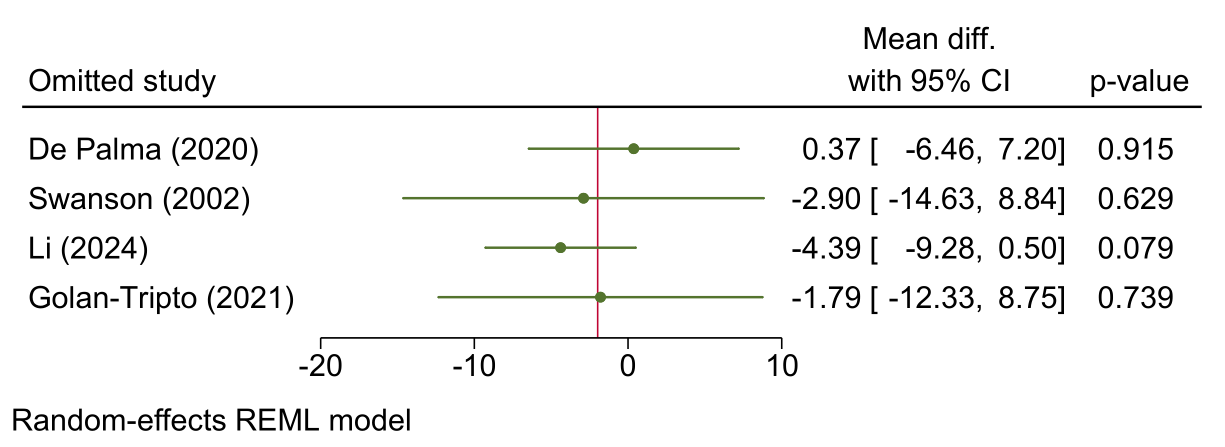

Supplement: Supplementary file 1 [file jcm-13-05652-s001.zip › Figure S5.tiff]

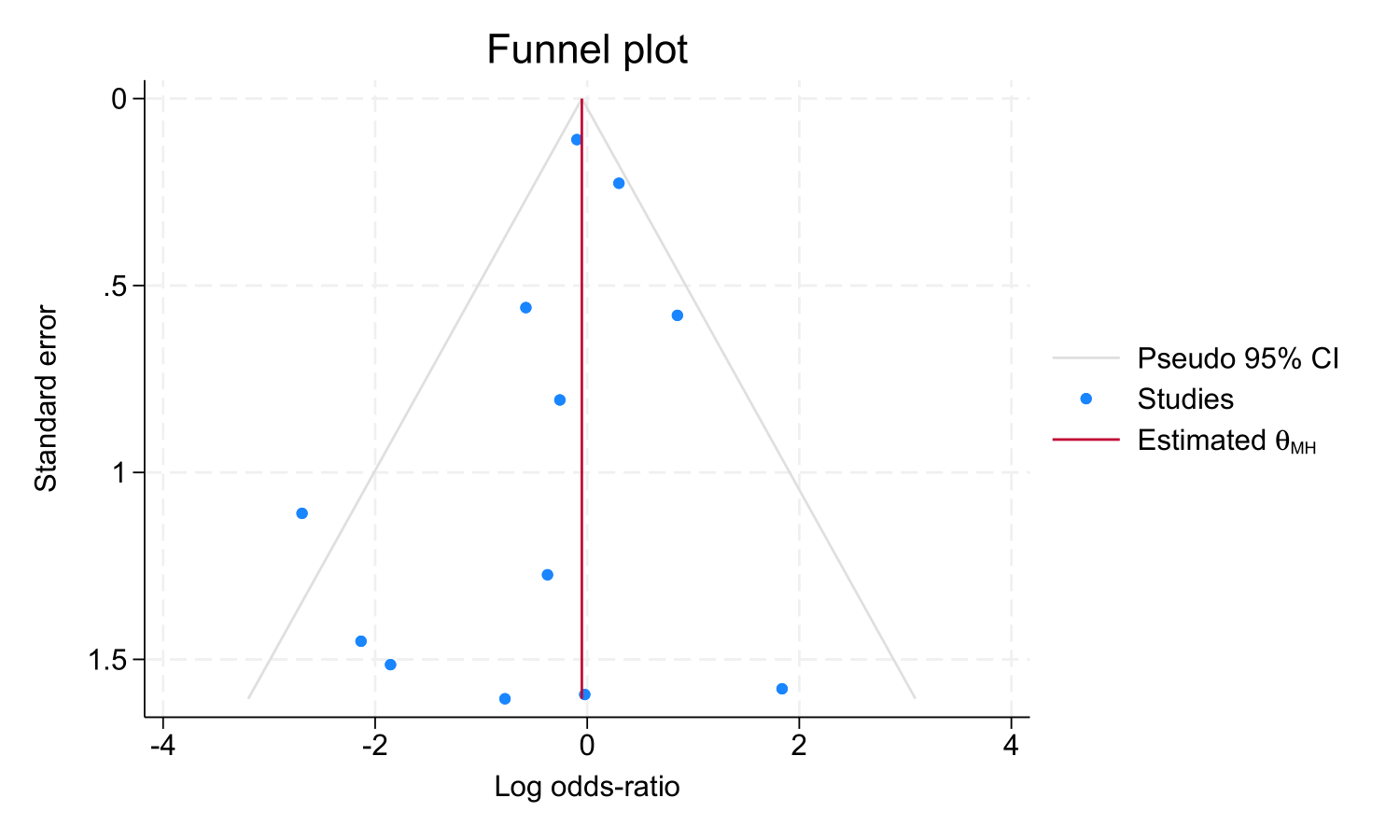

Supplement: Supplementary file 1 [file jcm-13-05652-s001.zip › Figure S6.tiff]

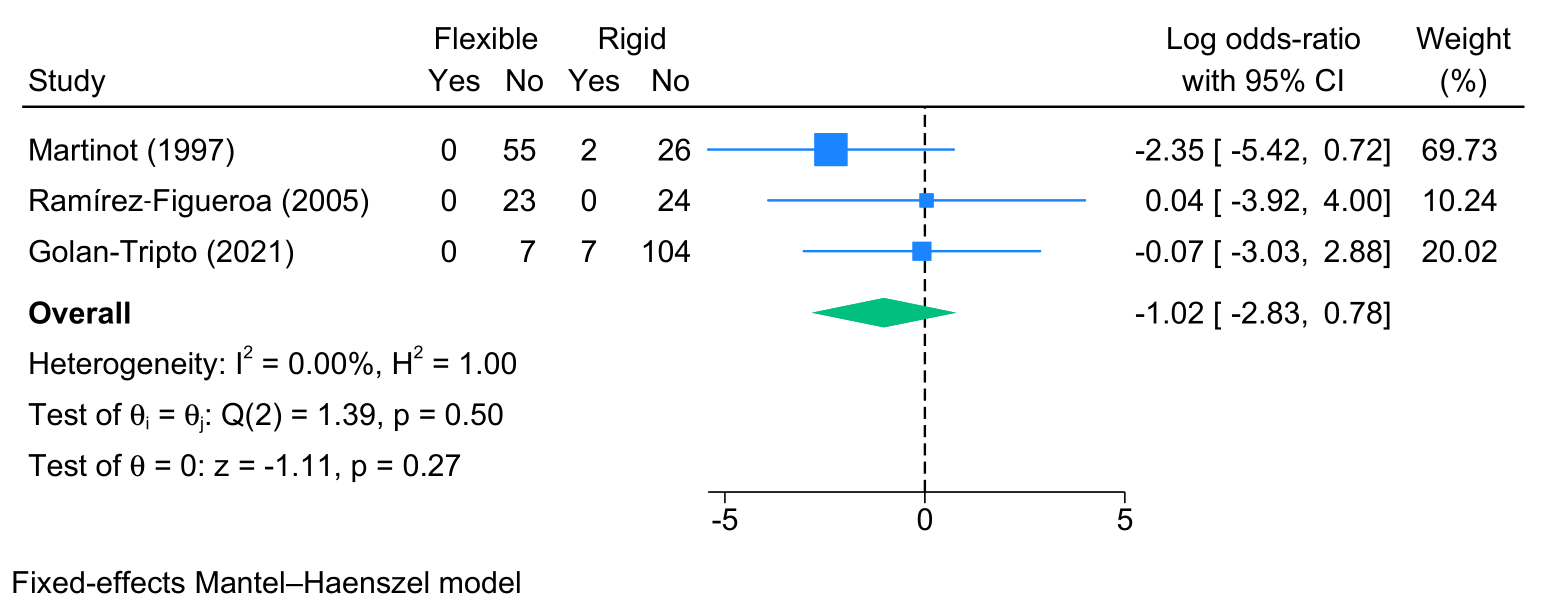

Supplement: Supplementary file 1 [file jcm-13-05652-s001.zip › Figure S7.tiff]

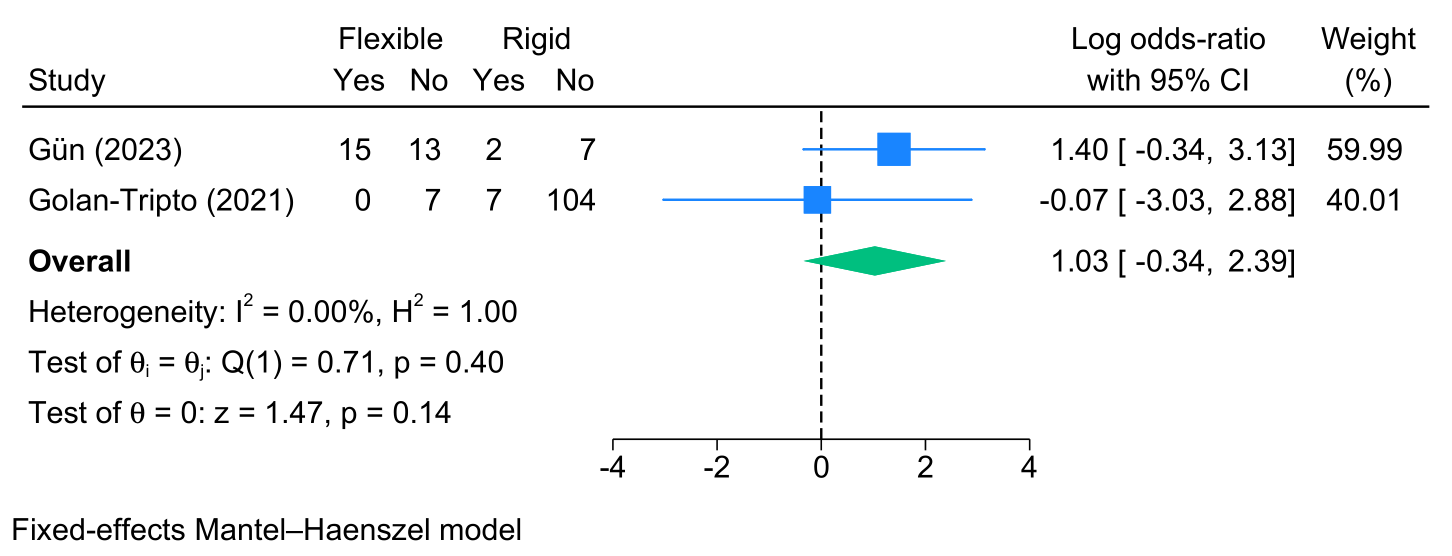

Supplement: Supplementary file 1 [file jcm-13-05652-s001.zip › Figure S8.tiff]
